# Prevalence of neutropenia in US residents: a population based analysis of NHANES 2011–2018

**DOI:** 10.1186/s12889-023-16141-5

**Published:** 2023-06-28

**Authors:** Jing Zhou, Nan Zhou, Qing Liu, Zhi-Ping Xie, Yun Xu, Si-Cheng Dai, Juan Lu, Zheng-Yang Bao, Li-Da Wu

**Affiliations:** 1 Department of Infectious Diseases, Affiliated Wuxi Fifth Hospital of Jiangnan University, The Fifth People’s Hospital of Wuxi, Wuxi, 214065 China; 2 Department of Nursing, Huadong Sanatorium, Wuxi, 214065 China; 3 Department of Anesthesiology, Huadong Sanatorium, Wuxi, 214065 China; 4 Department of Internal Medicine, Wuxi Maternity and Child Health Care Hospital, Women’s Hospital of Jiangnan University, Jiangnan University, Wuxi, 214002 China; 5 Clinical Medical College of Nanjing Medical University, Nanjing, 210029 China

**Keywords:** Neutropenia, Neutrophil, NHANES, Cross-sectional study

## Abstract

**Aims:**

Neutrophils play a pivotal in immunity and inflammation. We aim to investigate the prevalence of neutropenia in the United States.

**Methods:**

In this cross-sectional study, participants from the National Health and Nutrition Examination Survey (NHANES) (2011–2018) were enrolled. Demographic information, hematologic measurements, smoking status of all participants were collected for all participants. All statistical analyses were performed utilizing the NHANES survey weights. Covariate-adjusted linear regression was used to compare hematologic indices in different population grouped by age, sex, ethnicity, and smoking. We also employed multivariate-logistic regression to estimate the weighted odds ratio with a 95% confidence interval and predict the neutropenia risk among.

**Results:**

32,102 participants from NHANES survey were included, represented 286.6 million multiracial population in the United States. Black participants had lower mean leukocyte count (mean difference (MD): 0.71 × 10^9^/L; *P* < 0.001) and lower neutrophil count (MD: 0.83 × 10^9^/L; *P* < 0.001) compared with white participants after adjusting for age and sex. Furthermore, t a notable observation was the significant downward shift in the distribution curves of leukocyte count and neutrophil count among black participants. Smokers had a higher mean leukocyte count (MD: 1.10 × 10^9^ cells/L; *P* < 0.001) and a higher mean neutrophil count (MD: 0.75 × 10^9^ cells/L; *P* < 0.001) comparing with nonsmokers. The estimated prevalence of neutropenia was 1.24% (95% CI: 1.11 − 1.37%), which corresponds to approximately 35.5 million individuals in the United States. The prevalence of neutropenia in black participants was significantly higher than other races. Results of logistic regression analysis showed that black individuals, male individuals, and children younger than 5 years had a higher risk of neutropenia.

**Conclusions:**

Neutropenia is more common in the general population than we thought, especially in black individuals and children. More attention should be paid to neutropenia.

**Supplementary Information:**

The online version contains supplementary material available at 10.1186/s12889-023-16141-5.

## Introduction

Neutrophils, constituting the predominant type of granulocyte, account for approximately 40–70% of the total white blood cell population in the human body [1]. These cells originate from the bone marrow and exhibit lobulated or rod-shaped nuclei along with a large number of neutral granules in the cytoplasm that are neither basophilic nor acidophilic [2]. The cytoplasmic granules primarily consist of lysosomes containing a rich assortment of enzymes such as myeloperoxidase, lysozyme, alkaline phosphatase and acid hydrolase, which contribute to the processes of phagocytosis and cellular digestion [3, 4]. Neutrophils have chemotactic, phagocytic and bactericidal effects. As one of the most important lines of defense against invading pathogens, neutrophils have phagocytosis, which can remove foreign pathogens and engulf dead red blood cells. In addition, neutrophils can migrate across blood vessels to adjacent tissues and play an important role in immune responses [5].Neutropenia is defined as a neutrophil count below 1.5 × 109 cells/L [6]. Neutropenia can be classified as congenital neutropenia and acquired neutropenia [7], dominant mutations of autosomal in *ELANE* gene is the most common reason of congenital neutropenia, and virus, therapeutic radiation, and drugs can all lead to acquired neutropenia [8]. The primary clinical consequence of neutropenia is an elevated susceptibility to infections. The risk of infection is closely associated with the severity and duration of neutropenia, investigators demonstrated that a neutrophil count of less than 1 × 10^9^ cells/L increases the risk of infection by 36.7% [9], and prolonged exposure to this condition can further escalate the risk of severe infection. The prevalence of neutropenia exhibits significant variations among different racial groups. Asymptomatic neutropenia can be observed across all ethnic backgrounds, with previous studies reporting notable increases among populations from Middle Eastern, African, West Indian, and Caribbean [10–12]. Additionally substantial disparities exist in the incidence of neutropenia among various gender and age groups, with males and children considered to be at higher risk of developing neutropenia [13]. Although studies about neutropenia and its related risk factors have already existed, it is still necessary to do a detailed description of the prevalence of neutropenia in a large population [12, 14, 15].

National Health and Nutrition Examination Survey (NHANES) is a comprehensive program designed to assess the health and nutrition status of adults and children in the United States [16]. The extensive data within the NHANES database have been analyzed extensively, which is of great help in unraveling the etiologies, understanding the epidemiology, and searching for novel biomarkers of different diseases [17–19]. In this particular study, we specifically included participants from the four most recent consecutive circles of NHANES survey (2011–2018) to elucidate the features of prevalence of neutropenia within a large, multiracial population. Additionally, our objectives encompass exploring the potential influence of age, sex, ethnicity, and smoking on the prevalence of neutropenia.

## Methods

### Study Design and participants

NHANES survey, a continuous cross-sectional survey, conducted once every 2 years, by National Center for Health Statistics in the Centers for Disease Control and Prevention [20]. All participants enrolled in NHANES provided written informed consent, and the whole procedures were approved by the Institutional Review Board of the Centers for Disease Control and Prevention [21]. The method of stratified multistage probability sampling was adopted to screen out representative participants in NHANES survey, therefore, the population included in the present study can representing US adults and there was no need for a power analysis. Detailed methods are described in the NHANES website (http://www.cdc.gov/nchs/nhanes.htm). An analysis of the most recent four consecutive NHANES circles (2011–2012, 2013–2014, 2015–2016, and 2017–2018) was conducted in the present study. There was no need for randomization of blinding methods considering this is a cross-sectional study. The exclusion criteria of participants as followed: (1) missing hematologic measurements; (2) age ≤ 1 year old; (3) pregnant participants. This study is a population-based, cross-sectional study. The final population attrition was 42,866.

### Hematologic measurements and covariates

After at least 8 h of an overnight fast, 5 mL whole blood samples were collected from participants through venipuncture and anticoagulated by K3 EDTA to examine hematologic measurements based on Coulter MAXM counter (Beckman Coulter, Miami, Florida). Neutropenia was defined as a neutrophil count below 1.0 × 10^9^ cells/L. Demographic information, including age, sex, and race/ethnicity, was obtained through the administration of comprehensive questionnaires. Smoking status were determined from the health questionnaires. Age (years) was initially utilized as a continuous variable and then age groups were based on strata that are commonly used in highly age-stratified NHANES analyses [22]. Sex was categorized as male or female. Ethnicity was classified as non-Hispanic White, non-Hispanic Black, American Mexican, and others. Smoking status was categorized as either yes or no.

### Statistical methods

We followed the recommendation by the NHANES analytic and reporting guidance in the present study [23]. To reduce bias induced by post-stratification, non-response, and oversampling, stratified multistage probability sampling was adopted in NHANES. Primary sampling unit and specific sampling weight were assigned to every participant to ultimately produce representative estimates in the nation-wide. WTMEC2YR represent the survey weight for each participant who examined hematologic measurements in a Mobile Exam Center. Considering the most recent four consecutive cycles of NHAES after 2010 were analyzed, the finally 8-years weight was calculated as the followed formula: $WTMEC8YR=\frac{1}{9}\times WTMEC2YR$. The estimation of the representative population was derived by applying the 8-years survey weight in statistical analysis conducted in this study. Continuous variables were presented as the weighted mean (95% confidence interval (CI)), and categorical variables were represented with proportions. Comparisons of hematologic measurements in different groups was conducted using linear regression after adjusting for age and sex. The distribution of leukocyte and neutrophil counts, as well as the prevalence of neutropenia, were analyzed across various population subgroups defined by age, sex, and ethnicity. Furthermore, the proportions of participants with a neutrophil count < 1.0 × 10^9^ cells/L, and proportion of participants with a neutrophil count of 1.0–1.5 × 10^9^ cells/L were also analyzed. Moreover, mean neutrophil and leukocyte counts in the smoking group and nonsmoking group were compared in linear regression after adjusting for age, sex, and ethnicity. We adopted Logistic regression to generate weighted odds ratio with a 95% confidence interval to predict the risk of neutropenia. A two-sided *P* value < 0.05 was considered statistically significant. All statistical analyses were performed using R software (R Core Team, 2022; version 4.1.6).

## Results

### Study Population and hematologic measurements

A total of 32,102 participants with hematologic measurements from NHANES (2011–2018) was enrolled in the present study, which represented 286.6 million multiracial population in the United States. 3807 participants were excluded considering missing hematologic indices, of whom 27% were white participants, 30% were black participants, 13% were Mexican American participants, and 30% were other races; children ages 5 years or younger accounted for 36%, 30% were participants 6 to 18 years of age, adults 18 years or older accounted for 34%. Comparing with white participants, there was a lower mean leukocyte counts (mean difference (MD): 0.71 × 10^9^/L; *P* < 0.001), lower neutrophil counts (MD: 0.83 × 10^9^/L; *P* < 0.001), but higher lymphocyte counts (MD: 0.14 × 10^9^/L; *P* < 0.001) in black participants. Mexican Americans showed higher leukocyte counts (MD: 0.28 × 10^9^/L; *P* < 0.001), similar neutrophil counts (MD: 0.03 × 10^9^/L; *P* = 0.36), and higher lymphocyte counts (MD: 0.27 × 10^9^/L; *P* < 0.001) comparing with white participants. Participants from other races had similar leukocyte counts (MD: 0.28 × 10^9^/L; *P* < 0.001), lower neutrophil counts (MD: 0.13 × 10^9^/L; *P* = 0.36), and higher lymphocyte counts (MD: 0.15 × 10^9^/L; *P* < 0.001) compared with white participants. Furthermore, we conducted linear regression analysis to compare the hematologic parameters among distinct populations, stratified by age, sex, and ethnicity (Table 1). Moreover, Table S1 and Table S2 showed the detailed distribution of hematologic indices in different ethnic groups for sex and age strata recommended by NHANES.

**Table 1:** Comparation of Hematologic Measurements Grouped by Age, Sex, and Ethnicity

Sex and age group	Leukocyte count, 10^9^ cells/L				Neutrophil count, 10^9^ cells/L				Lymphocyte count, 10^9^ cells/L			
	Blacks	Whites	Mexican Americans	Other races	Blacks	Whites	Mexican Americans	Other races	Blacks	Whites	Mexican Americans	Other races
Males < 18 y	6.39(6.22,6.56) ^***^	7.23(7.09,7.38)	7.70(7.55,7.85) ^***^	7.54(7.41,7.67) ^***^	2.88(2.77,3.00) ^***^	3.53(3.43,3.63)	3.85(3.71,3.98) ^***^	3.73(3.61,3.84) ^*^	2.63(2.55,2.71) ^***^	2.79(2.72,2.85)	2.92(2.84,3.01) ^*^	2.89(2.82,2.97) ^*^
Males ≥ 18 y	6.46(6.36,6.56) ^***^	7.29(7.18,7.41)	7.45(7.33,7.57) ^*^	7.22(7.08,7.35)	3.55(3.47,3.62) ^***^	4.34(4.27,4.42)	4.38(4.26,4.50) ^***^	4.20(4.09,4.31) ^*^	2.13(2.09,2.16) ^**^	2.07(2.01,2.14)	2.24(2.18,2.30) ^*^	2.16(2.13,2.19) ^*^
Females < 18 y	6.70(6.54,6.86) ^***^	7.44(7.27,7.60)	7.78(7.63,7.93) ^**^	7.61(7.45,7.77)	3.11(3.01,3.21) ^***^	3.73(3.60,3.87)	3.99(3.88,4.10) ^**^	3.83(3.72,3.93)	2.79(2.71,2.86)	2.85(2.77,2.94)	2.95(2.88,3.03)	2.94(2.86,3.02)
Females ≥ 18 y	6.87(6.70,7.04) ^***^	7.44(7.34,7.55)	7.78(7.63,7.94) ^***^	7.38(7.26,7.50)	3.76(3.68,3.84) ^***^	4.51(4.43,4.59)	4.70(4.57,4.82) ^**^	4.36(4.27,4.45) ^*^	2.37(2.23,2.52) ^**^	2.14(2.10,2.17)	2.33(2.29,2.37) ^***^	2.26(2.22,2.29) ^***^

Note: data was presented as means (95% credibility intervals). The numbers of males younger than age 18 years were 1347, 1451, 1071, and 1480 among black, white, Mexican-American participant, and other races, respectively, and the numbers of males age 18 years or older were 2303, 2303, 1482, and 2762, respectively. The numbers of females younger than age 18 years were 1268, 1293, 1173, and 1173 among black, white, Mexican-American participants, and other races, and the numbers of females age 18 years or older were 2522, 4029, 1572, and 3070, respectively. credibility interval. ^***^P value < 0.001, ** *P* value < 0.01, * *P* value < 0.05, compared with white individuals

### Distribution of leukocyte and Neutrophil Counts

**Fig. 1:**
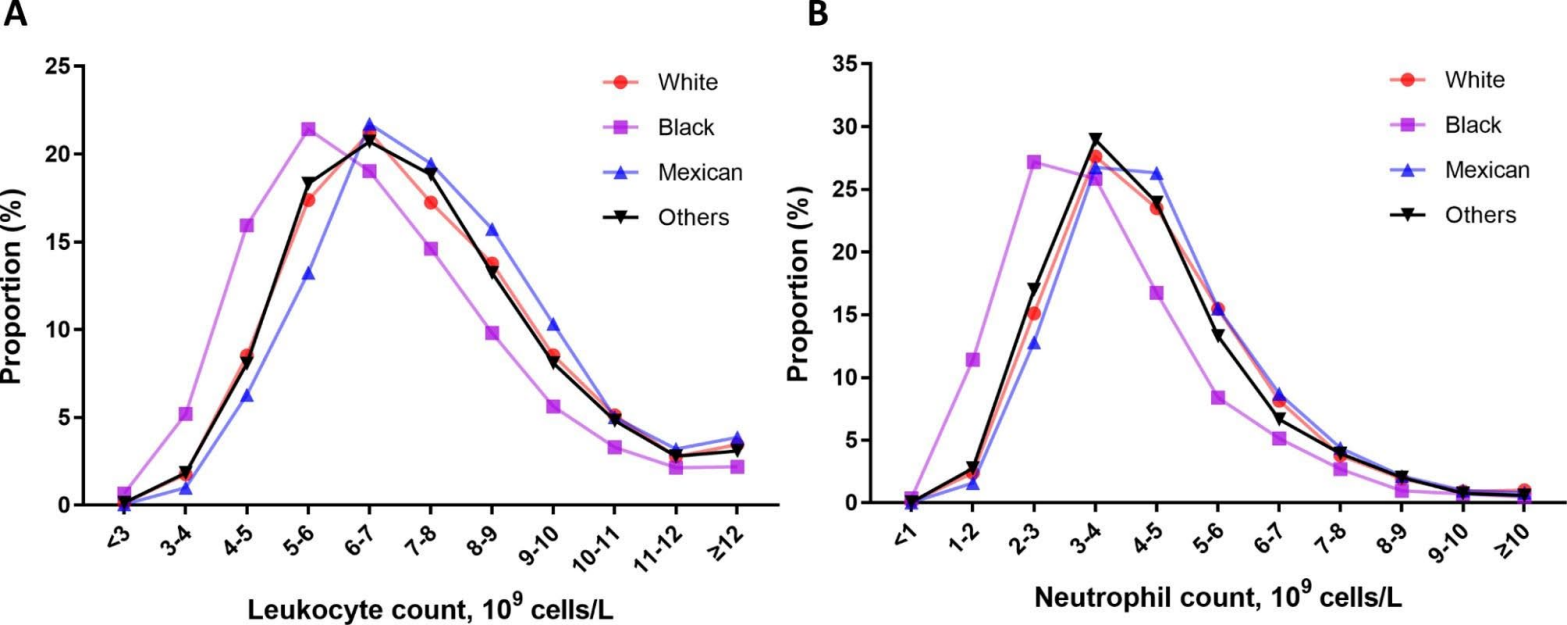
Distribution of population at different leukocyte (**A**) and neutrophil (**B**) counts

The distribution of population (age ≥ 18) grouped by leukocyte and neutrophil counts were shown in Table S3. We also visualized the results in Fig. 1. When comparing with White participants and American Mexican participants, there was a significant downward shift in Black participants. In White participants, American Mexican participants, and other ethnic participants, we did not observe shifts in either leukocyte or neutrophil counts. We also investigated the effect of smoking on the leukocyte and neutrophil counts using linear regression model after adjusting for age and sex. Smokers (former or now) had a higher mean leukocyte count (MD: 1.10 × 10^9^ cells/L) and a higher mean neutrophil count (MD: 0.75 × 10^9^ cells/L) comparing with nonsmokers (Table 2). Additionally, results of subgroup analysis grouped by ethnicity demonstrated that the impact of smoking on leukocyte count and neutrophil count was greatest in white participants and least in black participants (Table 2).

**Table 2:** Comparation of Leukocyte and Neutrophil Counts in Smokers and Nonsmokers

	Smokers	Nonsmokers	Mean Difference between Smokers and Nonsmokers	P value
**Leukocyte count, 10** ^**9**^ **cells/L**				
Overall	8.21(8.11, 8.32)	7.11(7.03, 7.19)	1.10(1.00, 1.21)	P < 0.001
Black	7.18(7.04, 7.31)	6.55(6.40, 6.69)	0.71(0.5, 0.92)	P < 0.001
White	8.44(8.31, 8.58)	7.13(7.03, 7.22)	1.29(1.15, 1.44)	P < 0.001
American Mexican	8.24(7.96, 8.52)	7.52(7.42, 7.61)	0.81(0.52, 1.09)	P < 0.001
Other	8.12(7.89, 8.35)	7.14(7.04, 7.24)	1.01(0.78, 1.24)	P < 0.001
**Neutrophil count, 10** ^**9**^ **cells/L**				
Overall	4.94(4.86, 5.02)	4.20(4.14, 4.26)	0.75(0.66, 0.83)	P < 0.001
Black	4.01(3.90, 4.11)	3.57(3.48, 3.65)	0.49(0.35, 0.63)	P < 0.001
White	5.14(5.03, 5.25)	4.27(4.20, 4.33)	0.87(0.75, 0.99)	P < 0.001
American Mexican	4.91(4.72, 5.11)	4.48(4.38, 4.57)	0.51(0.3, 0.72)	P < 0.001
Other	4.90(4.72, 5.08)	4.16(4.09, 4.24)	0.77(0.59, 0.95)	P < 0.001

Note: data was presented as percentage (95% credibility intervals). Comparisons of the means between smokers and nonsmokers, according to ethnicity and adjusted for age and sex in linear regression

### The prevalence of neutropenia

**Fig. 2:**
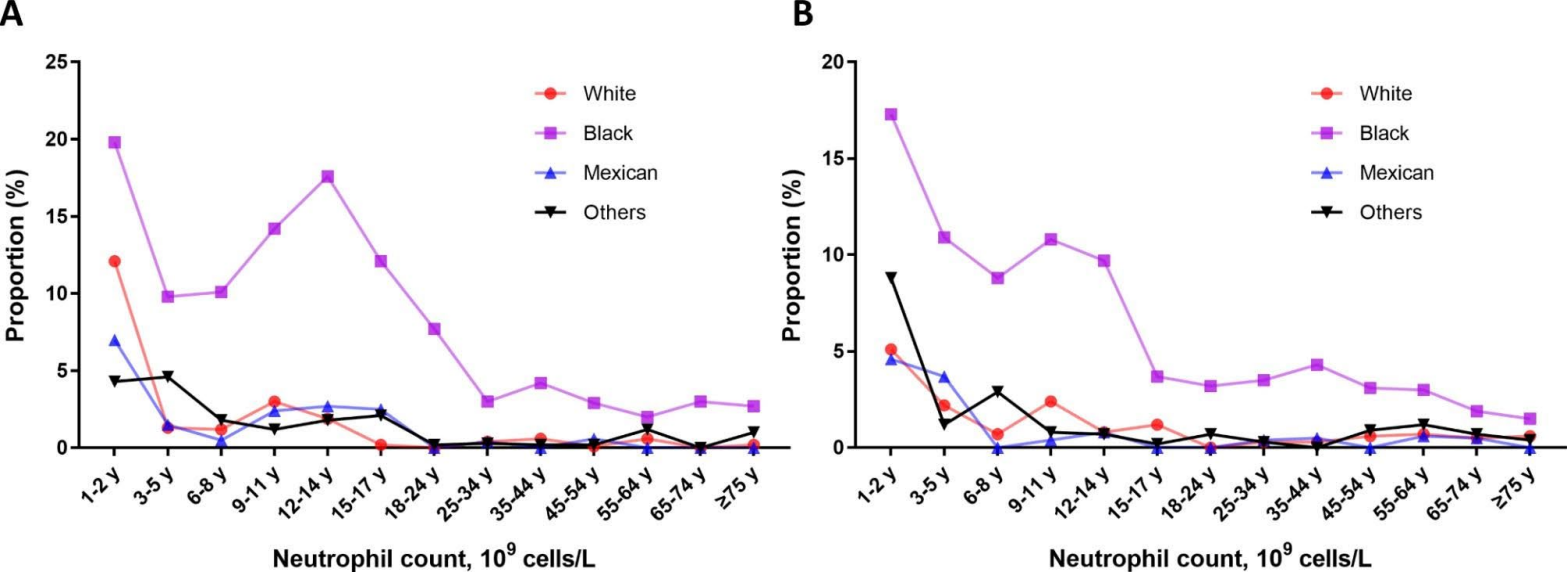
Proportion of male participants (**A**) and female participants (**B**) with neutropenia at different ages

Figure 2 and Table S4 show the prevalence of neutropenia in different populations grouped by age, sex, and ethnicity. 711 participants had neutropenia, and the weighted prevalence was 1.24% (95% CI: 1.11 − 1.37%), and represented 35.5 million residents in the United States. 5.36% (95% CI: 4.4 − 6.33%) black participants, 0.65% (95% CI: 0.46 − 0.85%) white participants, 0.76% (95% CI: 0.51 − 1.01%) American Mexican participants, and 0.84% (95% CI: 0.63 − 1.05%) participants from other races had neutropenia. The proportion of participants with neutropenia was higher in male individuals compared with female individuals across ethnic groups, 6.29% (95% CI: 5.00 − 7.59%) vs. 4.57% (95% CI: 3.59 − 5.54%) for black males and females, 0.66% (95% CI: 0.39 − 0.94%) vs. 0.64% (95% CI: 0.33 − 0.95%) for white males and females, 0.90% (95% CI: 0.54 − 1.27%) vs. 0.61% (95% CI: 0.33 − 0.90%) for American Mexican males and females, 0.86% (95% CI: 0.55 − 1.17%) vs. 0.82% (95% CI: 0.54 − 1.11%) for other ethnic males and females. Children younger than 5 years were more like to have neutropenia. Black participants were more likely to have neutropenia among every age and sex subgroup. There was no significant difference in the prevalence of neutropenia found among white participants, American Mexican participants, and other races.

### The Association between Age, Sex, ethnicity and Neutropenia

Furthermore, we conducted multivariable logistic regression analysis to examine the prevalence trend of neutropenia while adjusting for age, sex, and ethnicity. Our results revealed that Black individuals exhibited a higher likelihood of experiencing neutropenia when compared to white individuals (odds ratio: 5.48–10.02; *P <* 0.001), and the risk of neutropenia was similar in American Mexican individuals, individuals from other races, and white individuals. In addition, male individuals were more likely to have neutropenia than female individuals (odds ratio: 1.16–1.56; *P <* 0.05). Moreover, the prevalence of neutropenia was found to be higher during childhood and adolescence (Table 3). Notably, no significant interactions were observed between and ethnicity and sex (P = 0.99) or between ethnicity and age (P = 0.41). We also found that 86% of participants with neutropenia had a neutrophil count more than 1.0 × 10^9^ cells/L. 12.5% of back participants, 14.1% of white participants, 17.5% of American Mexican participants, and 18.1% participants from other races with neutropenia had neutrophil count less than 1.0 × 10^9^ cells/L. Moreover, 4.7% black participants, 0.56% white participants, 0.63% American Mexican participants, and 0.69% participants from other races had neutrophil counts less than 1.0 × 10^9^ cells/L in the whole population. The prevalence of neutrophil counts less than 1.0 × 10^9^ cells/L in different population grouped by age was shown in Table 4.

**Table 3:** Prevalence of Neutropenia in the United States

Variable	Prevalence, % (95% CI)	
**Ethnicity**		
Black	5.36(4.72, 6.00)	
White	0.65(0.48, 0.82)	
Mexican	0.76(0.55, 0.98)	
Other	0.84(0.66, 1.02)	
**Sex**		
Male	1.34(1.12, 1.56)	
Female	1.15(0.95, 1.34)	
**Age**		
1–2 y	9.13(7.18, 11.08)	
3–5 y	3.39(2.46, 4.33)	
6–8 y	2.30(1.61, 2.99)	
9–11 y	3.49(2.54, 4.44)	
12–14 y	3.17(2.26, 4.07)	
15–17 y	1.88(1.16, 2.60)	
18–24 y	0.83(0.53, 1.12)	
25–34 y	0.67(0.33, 1.01)	
35–44 y	0.80(0.41, 1.19)	
45–54 y	0.65(0.27, 1.03)	
55–64 y	0.90(0.51, 1.29)	
65–74 y	0.44(0.10, 0.77)	
≥ 75 y	0.55(0.29, 0.82)	

**Table 4:** Proportion of Participants with Neutropenia with Neutrophil Counts of 1.0–1.5 × 10^9^ Cells/L and Less than 1.0 × 10^9^ Cells/L

Age group	Participants with a neutrophil count < 1.0 × 10^9^ cells/L, n (%) [95% CI]	Participants with a neutrophil count of 1.0–1.5 × 10^9^ cells/L, n (%) [95% CI]
1–2 y	12(3.34)[1.86–4.83]	48(15.13)[10.80-19.46]
3–5 y	4(0.87)[-0.02-1.77]	40(9.45)[6.83–12.07]
6–8 y	3(0.76)[-0.13-1.65]	42(8.70)[5.74–11.66]
9–11 y	15(2.58)[1.30–3.86]	52(9.85)[6.63–13.08]
12–14 y	8(1.81)[0.33–3.29]	53(11.94)[8.14–15.74]
15–17 y	5(1.26)[0.30–2.22]	30(6.95)[4.31–9.59]
18–24 y	4(0.42)[-0.01-0.86]	33(4.95)[2.95–6.96]
25–34 y	1(0.24)[-0.23-0.71]	22(3.02)[1.91–4.12]
35–44 y	4(0.64)[0.00-1.29]	23(3.64)[2.00-5.28]
45–54 y	1(0.30)[-0.28-0.87]	22(2.66)[1.59–3.74]
55–64 y	3(0.36)[-0.05-0.76]	23(2.22)[1.33–3.11]
65–74 y	2(0.37)[-0.15-0.90]	14(2.03)[1.02–3.05]
≥ 75 y	0(0.00)[0.00–0.00]	8(1.98)[0.51–3.45]

## Discussion

Neutrophil plays a pivotal role in immune response and inflammation. Upon pathogenic invasion, chemokine activation prompts neutrophils to migrate from capillaries to the site of injury, enabling them to engage in pathogen phagocytosis [24]. Concurrently,neutrophils contain a significant quantity of lysosomal enzymes, which can also decompose bacteria and tissue debris phagocytosed into cells, so as to avoid the spread of infection in the body [25]. Neutropenia, characterized by a neutrophil counts below 1.5 × 10^9^ cell/L, heightens the susceptibility to infection. Based on a large multiracial population from NHANES survey in the United States, our results showed that 1.24% population in the United States had neutropenia, approximately 35.5 million nationwide. Besides, the proportion of black individuals with neutropenia were significantly higher than participants from other races. Male participants were more likely to have neutropenia than female participants, and children younger than 5 years had a higher risk of neutropenia than adults.

The underlying mechanisms of congenital neutropenia remain largely elusive [26]. Some researchers have postulated that neutropenia may occur due to a dysfunction in the release of mature granulocytes from the bone marrow. A recent study enrolled 30 patients with congenital neutropenia (14 black participants and 16 white participants) and demonstrated that the bone marrow of black participants released fewer neutrophils than that of white participants through bone marrow biopsies [27]. Interestingly, no disparities were observed in terms of bone marrow cellularity and the degree of myeloid maturation between Black and White individuals following hydrocortisone treatment, suggesting similar bone marrow hematopoietic function. The similar increase in neutrophil count was also observe in black and white participants. On the other hand, Bain et al. demonstrated that the increment of neutrophils was significantly higher in normal individuals compared with individuals with congenital neutropenia after receiving the injection of hydrocortisone, also indicating similar hematopoietic function but decreased marrow response in Yemenite Jews patients with neutropenia [28]. Nevertheless, it is worth emphasizing that ethnicity exerts a significant influence on the prevalence of congenital neutropenia, necessitating further studies investigating neutropenia among diverse racial populations. Dominant mutations of autosomal in *ELANE* gene is the most common reason of congenital neutropenia [29]. With the development of genomics and sequencing technology, more and more attention has been paid to the correlation between changes in the expression of genes and incidence of neutropenia in recent years [30–32]. Studies have also revealed significant alterations in the that expression of genes associated with leukocyte migration and hematopoietic stem cells mobilization significantly changed among individuals with neutropenia [29, 33, 34]. To gain a better understanding of the prevalence characteristics of neutropenia, we enrolled participants from recent four consecutive circles of NHANES (2011–2012, 2013–2014, 2015–2016, 2017–2018) to carry out this population-based cross-sectional study.

As mentioned before, the prevalence of neutropenia in different races are significantly different, in the present study we mainly focus on three major ethnicities in the United States, including non-Hispanic White, non-Hispanic Black, American Mexican. The first report on the difference of neutropenia risk between black and white individuals was 1941, investigators found that residents of African descent in the United States had lower leukocyte counts and firstly thought it was related to nutriture, and then found that was a result of neutropenia [35]. Consequently, Freedman et al. carried out a cross-sectional study and reported that the difference of the neutrophil count was more significant in black male individuals based on a large population in the United States [36]. At the same time, in the United Kingdom, Bain et al. also demonstrated that residents of African descent had lower neutropenia count [28]. In this study, we observed a significant downward shift (approximately 1.0 × 10^9^ cells/L) in distribution of both leukocyte count and neutrophil count among black participants. The leukocyte count and neutrophil count exhibited similar distributions among white participants, American Mexican participants, and participants from other racial backgrounds. Consistent with previous research, the present study observed a higher risk of neutropenia among black participants. Of note, our study revealed a noteworthy incidence of neutropenia in black children, 19.8% of black children (age 1–2 years) and 9.8% of black children (age 3–5 years) had neutropenia.

Leukocyte count and neutrophil count are different at different ages. Investigators have revealed that the leukocyte count were highest at birth, and then decreased rapidly in the first six month, and the prevalence of neutropenia is higher in children younger than 9 years compared with adults [37]. Chronic and severe neutropenia in children can have serious consequences, it has been reported that the incidence of infection in children with neutropenia was 44.2%, and the incidence of severe infection ranged from 9.6 − 11.9% [38]. Children with congenital neutropenia often have other clinical manifestations in addition to neutropenia.

Some children are complicated with abnormal liver function, nephrotic syndrome, hearing loss, ventricular septal defect, iron deficiency anemia, etc. [39]. Consistent with previous studies, results of our study also showed that children younger than 5 years were more like to have neutropenia. Lyall et al. reported that the prevalence of neutropenia in preterm infants is higher than that in full-term infants, which may be related to the imbalance of immune regulation caused by the immature immune system [40]. Therefore, more attention should be paid to neutropenia, especially in children younger than 5 years.

Furthermore, we investigated the influence of smoking on the prevalence of neutropenia and found that smokers had higher leukocyte and neutrophil counts than those of participants who never smoked. This conclusion is consistent with an earlier retrospective study performed by Aghaji et al., they enrolled 5850 Nigerian participants and found that the leukocyte count was higher than nonsmokers [41]. Another study indicated a higher likelihood of neutropenia in male individuals compared to females. Consistent with these findings, we observed difference in leukocyte and neutrophil count between male and female participants, and our multivariable logistic regression analysis confirmed a higher risk of neutropenia among males [42]. Our results suggest that neutropenia is more common in the general population than we thought. The need for a thorough diagnostic evaluation of neutropenia requires consideration of multiple factors such as clinical symptoms, age, sex, race, and smoking status.

There are several advantages and limitations of our study. First, it was adequate to provide reliable conclusion and precise statistical power considering the large-scale sample size included; second, we adopted the most recent data from NHANE survey, and all of the statistical processes were weighted for a more objective and comprehensive study of the prevalence of neutropenia in the United States. However, it is important to acknowledge several limitations of this study. Firstly, the causal associations could not be determined considering the research type of cross-sectional study, more prospective studies are needed to determine the exact risk factors of neutropenia. Secondly, the self-reported covariates obtained from the NHANES database may introduce potential subjective bias. Thirdly, there are large ethnic differences in diet, physical activity, genetic variants, lipid metabolism, and susceptibility to cardiovascular disease. Consequently, whether the conclusion in the present study based on US participants could be applicable to other populations need to be further explored in the future work.

## Conclusion

Our results highlighted that the prevalence of neutropenia in the United States. Black participants had lower leukocyte and neutrophil counts and were more likely to develop neutropenia. Besides, more attention should be paid to neutropenia in children. To evaluate neutropenia, not only clinical symptoms, but also multiple factors including ethnicity, age, and sex need to be considered. Healthcare professionals may need to be of these factors and incorporate them into their evaluation of neutropenic patients. While our study provides valuable insights, there is still much to be explored in the field of neutropenia. More prospective studies are needed to ascertain risk factors for neutropenia.

## Electronic supplementary material

Below is the link to the electronic supplementary material.

Supplementary Material 1

Supplementary Material 2

Supplementary Material 3

Supplementary Material 4

## Data Availability

Publicly available datasets were analyzed in this study. All the raw data used in this study are derived from the public NHANES data portal (https://wwwn.cdc.gov/nchs/nhanes/analyticguidelines.aspx).
