# Supplementary material for: Prevalence of neutropenia in US residents: a population based analysis of NHANES 2011–2018
Source: BMC Public Health. 2023 Jun 28;23:1254. doi: 10.1186/s12889-023-16141-5 (PMC10308693; doi:10.1186/s12889-023-16141-5)
Supplement: Supplementary file 1 — Supplementary Material 1 [file 12889_2023_16141_MOESM1_ESM.docx]

**Table S1. Detailed Comparation of Hematologic Measurements Grouped by Age, Sex, and Ethnicity**

| **Sex and age group** | **Participants, n** | **Leukocyte count,**  **109 cells/L** | **Neutrophil count, 109 cells/L** | **Lymphocyte count, 109 cells/L** | **Hemoglobin level,**  **g/L** | **Platelet count,**  **109 cells/L** |
| --- | --- | --- | --- | --- | --- | --- |
| **Black participants** |  |  |  |  |  |  |
| All | 7440 | 6.65(6.56, 6.75) ^***^ | 3.51(3.45, 3.56) ^***^ | 2.37(2.30, 2.43) ^***^ | 13.18(13.11, 13.24) ^***^ | 253.01(250.33, 255.68) ^***^ |
| Males <18 y | 1347 | 6.39(6.22, 6.56) ^***^ | 2.88(2.77, 3.00) ^***^ | 2.63(2.55, 2.71) ^***^ | 13.00(12.89, 13.10) ^***^ | 279.20(273.36, 285.05) |
| Males ≥18 y | 2303 | 6.46(6.36, 6.56) ^***^ | 3.55(3.47, 3.62) ^***^ | 2.13(2.09, 2.16) ^**^ | 14.35(14.28, 14.41) ^***^ | 221.72(219.09, 224.36) |
| Females <18 y | 1268 | 6.70(6.54, 6.86) ^***^ | 3.11(3.01, 3.21) ^***^ | 2.79(2.71, 2.86) | 12.37(12.28, 12.45) ^***^ | 292.88(287.68, 298.07) ^***^ |
| Females ≥18 y | 2522 | 6.87(6.70, 7.04) ^***^ | 3.76(3.68, 3.84) ^***^ | 2.37(2.23, 2.52) ^**^ | 12.50(12.42, 12.58) ^***^ | 260.00(256.54, 263.46) ^***^ |
| **White participants** |  |  |  |  |  |  |
| All | 10711 | 7.37(7.28, 7.45) | 4.30(4.24, 4.36) | 2.22(2.18, 2.26) | 14.18(14.13, 14.22) | 242.86(240.33, 245.39) |
| Males <18 y | 1451 | 7.23(7.09, 7.38) | 3.53(3.43, 3.63) | 2.79(2.72, 2.85) | 13.69(13.58, 13.79) | 273.97(269.19, 278.75) |
| Males ≥18 y | 3938 | 7.44(7.27, 7.60) | 3.73(3.60, 3.87) | 2.85(2.77, 2.94) | 13.22(13.14, 13.30) | 281.40(277.30, 285.51) |
| Females <18 y | 1293 | 7.29(7.18, 7.41) | 4.34(4.27, 4.42) | 2.07(2.01, 2.14) | 15.11(15.05, 15.17) | 223.70(221.13, 226.28) |
| Females ≥18 y | 4029 | 7.44(7.34, 7.55) | 4.51(4.43, 4.59) | 2.14(2.10, 2.17) | 13.57(13.52, 13.62) | 247.78(244.79, 250.77) |
| **Mexican American participants** |  |  |  |  |  |  |
| All | 5298 | 7.65(7.55, 7.75) ^***^ | 4.34(4.26, 4.41) | 2.49(2.45, 2.53) ^***^ | 13.94(13.88, 14.01) ^***^ | 257.12(254.44, 259.81) ^***^ |
| Males <18 y | 1071 | 7.70(7.55, 7.85) ^***^ | 3.85(3.71, 3.98) ^***^ | 2.92(2.84, 3.01) ^*^ | 13.64(13.52, 13.76) | 276.44(272.06, 280.82) |
| Males ≥18 y | 1482 | 7.78(7.63, 7.93) ^*^ | 3.99(3.88, 4.10) ^***^ | 2.95(2.88, 3.03) ^*^ | 13.02(12.95, 13.10) ^***^ | 288.54(284.17, 292.91) ^***^ |
| Females <18 y | 1173 | 7.45(7.33, 7.57) ^**^ | 4.38(4.26, 4.50) ^**^ | 2.24(2.18, 2.30) | 15.27(15.19, 15.35) ^***^ | 230.80(227.16, 234.44) ^*^ |
| Females ≥18 y | 1572 | 7.78(7.63, 7.94) ^***^ | 4.70(4.57, 4.82) ^**^ | 2.33(2.29, 2.37) ^***^ | 13.12(13.04, 13.20) ^***^ | 260.52(257.20, 263.83) ^***^ |
| **Other ethnic participants** |  |  |  |  |  |  |
| All | 8653 | 7.36(7.28, 7.45) | 4.17(4.09, 4.24) ^**^ | 2.37(2.35, 2.40) ^***^ | 13.83(13.77, 13.88) ^***^ | 252.92(250.90, 254.95) ^***^ |
| Males <18 y | 1480 | 7.54(7.41, 7.67) ^***^ | 3.73(3.61, 3.84) ^*^ | 2.89(2.82, 2.97) ^*^ | 13.48(13.35, 13.60) ^**^ | 280.75(276.91, 284.60) ^*^ |
| Males ≥18 y | 2762 | 7.61(7.45, 7.77) | 3.83(3.72, 3.93) ^*^ | 2.94(2.86, 3.02) ^*^ | 12.92(12.84, 13.01) ^***^ | 288.53(284.55, 292.52) ^**^ |
| Females <18 y | 1341 | 7.22(7.08, 7.35) | 4.20(4.09, 4.31) | 2.16(2.13, 2.19) | 14.97(14.90, 15.05) ^***^ | 229.33(226.33, 232.34) ^***^ |
| Females ≥18 y | 3070 | 7.38(7.26, 7.50) | 4.36(4.27, 4.45) ^*^ | 2.26(2.22, 2.29) ^***^ | 13.13(13.06, 13.19) ^***^ | 256.49(253.36, 259.61) ^***^ |

Note: data was presented as means (95% credibility intervals). ^***^ *P* value<0.001, ** *P* value<0.01, * *P* value<0.05, compared with white individuals.
