# Supplementary material for: Prevalence of neutropenia in US residents: a population based analysis of NHANES 2011–2018
Source: BMC Public Health. 2023 Jun 28;23:1254. doi: 10.1186/s12889-023-16141-5 (PMC10308693; doi:10.1186/s12889-023-16141-5)
Supplement: Supplementary file 2 — Supplementary Material 2 [file 12889_2023_16141_MOESM2_ESM.docx]

**Table S2. Hematologic Measurements Grouped by Age Strata and Ethnicity**

| **Age group** | | | **Participants, n** | | | **Leukocyte count, 10^9^ cells/L** | | | **Neutrophil count, 10^9^ cells/L** | **Lymphocyte count, 10^9^ cells/L** | | | **Hemoglobin level,**  **g/L** | | | **Platelet count,**  **10^9^ cells/L** |
| --- | --- | --- | --- | --- | --- | --- | --- | --- | --- | --- | --- | --- | --- | --- | --- | --- |
| **Black participants** | | |  | | |  | | |  |  | | |  | | |  |
| 1-2 y | | | 330 | | | 7.97(7.70, 8.24) | | | 2.68(2.51, 2.86) | 4.29(4.12, 4.46) | | | 11.95(11.85, 12.05) | | | 334.25(325.92, 342.58) |
| 3-5 y | | | 433 | | | 7.16(6.94, 7.39) | | | 2.96(2.80, 3.12) | 3.29(3.17, 3.40) | | | 12.13(12.02, 12.23) | | | 325.59(318.39, 332.78) |
| 6-8 y | | | 496 | | | 6.71(6.49, 6.93) | | | 3.09(2.93, 3.24) | 2.75(2.66, 2.84) | | | 12.42(12.34, 12.50) | | | 295.44(288.60, 302.28) |
| 9-11 y | | | 515 | | | 6.12(5.92, 6.31) | | | 2.82(2.69, 2.96) | 2.46(2.39, 2.53) | | | 12.62(12.53, 12.72) | | | 281.99(275.87, 288.10) |
| 12-14 y | | | 429 | | | 6.10(5.91, 6.28) | | | 3.01(2.86, 3.16) | 2.30(2.23, 2.38) | | | 13.04(12.93, 13.16) | | | 264.81(259.09, 270.54) |
| 15-17 y | | | 412 | | | 6.13(5.91, 6.34) | | | 3.22(3.06, 3.39) | 2.15(2.10, 2.21) | | | 13.38(13.21, 13.55) | | | 250.42(244.02, 256.82) |
| 18-24 y | | | 678 | | | 6.48(6.31, 6.64) | | | 3.55(3.42, 3.67) | 2.18(2.13, 2.23) | | | 13.47(13.33, 13.61) | | | 246.48(241.97, 250.99) |
| 25-34 y | | | 719 | | | 6.80(6.63, 6.97) | | | 3.76(3.63, 3.89) | 2.28(2.22, 2.34) | | | 13.47(13.33, 13.61) | | | 246.40(241.09, 251.71) |
| 35-44 y | | | 697 | | | 6.66(6.50, 6.83) | | | 3.70(3.57, 3.82) | 2.22(2.16, 2.28) | | | 13.16(13.02, 13.30) | | | 252.70(247.07, 258.32) |
| 45-54 y | | | 786 | | | 6.75(6.59, 6.91) | | | 3.70(3.59, 3.81) | 2.29(2.23, 2.36) | | | 13.41(13.28, 13.54) | | | 245.28(238.95, 251.61) |
| 55-64 y | | | 987 | | | 6.64(6.51, 6.76) | | | 3.64(3.54, 3.74) | 2.22(2.17, 2.28) | | | 13.46(13.35, 13.56) | | | 239.46(234.55, 244.36) |
| 65-74 y | | | 602 | | | 7.10(6.02, 8.17) | | | 3.55(3.40, 3.69) | 2.76(1.78, 3.73) | | | 13.15(13.00, 13.30) | | | 228.28(222.30, 234.25) |
| ≥75 y | | | 356 | | | 6.30(6.11, 6.49) | | | 3.67(3.50, 3.84) | 1.84(1.77, 1.91) | | | 12.53(12.37, 12.68) | | | 211.60(203.86, 219.34) |
| **White participants** | | |  | | |  | | |  |  | | |  | | |  |
| 1-2 y | | | 405 | | | 8.89(8.61, 9.17) | | | 3.12(2.94, 3.29) | 4.71(4.56, 4.87) | | | 12.47(12.37, 12.57) | | | 335.27(326.27, 344.27) |
| 3-5 y | | | 410 | | | 8.13(7.86, 8.39) | | | 3.60(3.43, 3.77) | 3.55(3.42, 3.68) | | | 12.69(12.58, 12.79) | | | 311.57(301.79, 321.36) |
| 6-8 y | | | 511 | | | 7.42(7.18, 7.66) | | | 3.65(3.45, 3.85) | 2.85(2.75, 2.95) | | | 13.07(12.98, 13.16) | | | 287.97(281.35, 294.59) |
| 9-11 y | | | 504 | | | 6.92(6.75, 7.08) | | | 3.49(3.38, 3.61) | 2.55(2.47, 2.63) | | | 13.41(13.31, 13.51) | | | 269.38(263.96, 274.80) |
| 12-14 y | | | 450 | | | 6.75(6.58, 6.91) | | | 3.59(3.47, 3.71) | 2.34(2.28, 2.40) | | | 14.00(13.84, 14.15) | | | 258.12(251.64, 264.60) |
| 15-17 y | | | 464 | | | 7.02(6.79, 7.24) | | | 3.96(3.77, 4.16) | 2.24(2.18, 2.31) | | | 14.16(14.03, 14.29) | | | 250.20(244.54, 255.85) |
| 18-24 y | | | 825 | | | 7.55(7.34, 7.76) | | | 4.47(4.30, 4.64) | 2.27(2.21, 2.32) | | | 14.51(14.40, 14.62) | | | 243.98(239.64, 248.32) |
| 25-34 y | | | 1213 | | | 7.60(7.46, 7.74) | | | 4.51(4.40, 4.61) | 2.27(2.22, 2.32) | | | 14.42(14.34, 14.50) | | | 241.74(237.57, 245.90) |
| 35-44 y | | | 1122 | | | 7.55(7.39, 7.71) | | | 4.56(4.44, 4.68) | 2.17(2.13, 2.21) | | | 14.40(14.30, 14.50) | | | 243.34(238.97, 247.71) |
| 45-54 y | | | 1170 | | | 7.39(7.22, 7.56) | | | 4.47(4.33, 4.60) | 2.11(2.05, 2.16) | | | 14.38(14.31, 14.46) | | | 241.83(237.59, 246.08) |
| 55-64 y | | | 1200 | | | 7.09(6.93, 7.26) | | | 4.27(4.15, 4.38) | 2.00(1.94, 2.06) | | | 14.39(14.29, 14.49) | | | 232.28(227.92, 236.63) |
| 65-74 y | | | 1096 | | | 7.05(6.89, 7.21) | | | 4.27(4.14, 4.39) | 1.92(1.87, 1.97) | | | 14.24(14.12, 14.35) | | | 227.33(223.08, 231.58) |
| ≥75 y | | | 1341 | | | 7.49(7.21, 7.77) | | | 4.55(4.43, 4.67) | 2.03(1.79, 2.27) | | | 13.64(13.55, 13.72) | | | 216.74(213.02, 220.47) |
|  | |  | | |  | | |  | | | |  | | |  |  |
| 1-2 y | | | 269 | | | 9.16(8.85, 9.46) | | | 3.19(3.01, 3.36) | 4.95(4.76, 5.14) | | | 12.38(12.28, 12.47) | | | 330.46(318.65, 342.27) |
| 3-5 y | | | 357 | | | 8.17(7.88, 8.45) | | | 3.80(3.60, 3.99) | 3.45(3.33, 3.57) | | | 12.64(12.54, 12.74) | | | 306.79(298.87, 314.72) |
| 6-8 y | | | 421 | | | 7.72(7.52, 7.93) | | | 4.00(3.84, 4.15) | 2.82(2.72, 2.92) | | | 13.04(12.94, 13.13) | | | 291.74(285.08, 298.39) |
| 9-11 y | | | 469 | | | 7.52(7.32, 7.71) | | | 3.92(3.77, 4.06) | 2.70(2.61, 2.79) | | | 13.36(13.27, 13.45) | | | 282.39(276.07, 288.72) |
| 12-14 y | | | 374 | | | 7.15(6.94, 7.37) | | | 3.89(3.73, 4.05) | 2.44(2.37, 2.52) | | | 13.82(13.66, 13.97) | | | 261.01(254.31, 267.71) |
| 15-17 y | | | 354 | | | 7.46(7.23, 7.70) | | | 4.38(4.18, 4.58) | 2.29(2.21, 2.36) | | | 14.21(14.05, 14.37) | | | 248.48(242.29, 254.68) |
| 18-24 y | | | 485 | | | 7.77(7.55, 7.99) | | | 4.65(4.46, 4.85) | 2.32(2.25, 2.38) | | | 14.49(14.34, 14.64) | | | 250.42(244.76, 256.08) |
| 25-34 y | | | 494 | | | 7.77(7.59, 7.95) | | | 4.63(4.48, 4.79) | 2.34(2.26, 2.43) | | | 14.38(14.23, 14.54) | | | 248.92(243.38, 254.46) |
| 35-44 y | | | 582 | | | 7.60(7.42, 7.78) | | | 4.48(4.34, 4.62) | 2.33(2.27, 2.39) | | | 14.09(13.93, 14.25) | | | 248.87(244.64, 253.11) |
| 45-54 y | | | 482 | | | 7.50(7.28, 7.73) | | | 4.48(4.31, 4.64) | 2.24(2.16, 2.31) | | | 14.16(14.00, 14.32) | | | 247.49(240.72, 254.25) |
| 55-64 y | | | 531 | | | 7.41(7.20, 7.62) | | | 4.35(4.20, 4.50) | 2.26(2.19, 2.33) | | | 14.37(14.27, 14.47) | | | 234.24(228.73, 239.75) |
| 65-74 y | | | 337 | | | 7.36(7.07, 7.66) | | | 4.45(4.23, 4.68) | 2.07(1.98, 2.16) | | | 13.76(13.54, 13.98) | | | 227.09(219.25, 234.94) |
| ≥75 y | | | 143 | | | 7.21(6.78, 7.65) | | | 4.54(4.20, 4.88) | 1.80(1.68, 1.93) | | | 13.24(13.02, 13.46) | | | 209.24(201.09, 217.39) |
|  | |  | | |  | | |  | | | |  | | |  |  |
| 1-2 y | | | 315 | | | 9.02(8.73, 9.31) | | | 3.11(2.93, 3.29) | 4.85(4.64, 5.05) | | | 12.32(12.24, 12.40) | | | 335.87(328.02, 343.72) |
| 3-5 y | | | 463 | | | 8.11(7.87, 8.35) | | | 3.74(3.56, 3.93) | 3.43(3.29, 3.58) | | | 12.55(12.45, 12.65) | | | 310.89(303.28, 318.49) |
| 6-8 y | | | 529 | | | 7.55(7.32, 7.78) | | | 3.71(3.54, 3.87) | 2.92(2.84, 3.00) | | | 12.84(12.74, 12.94) | | | 295.21(288.64, 301.78) |
| 9-11 y | | | 530 | | | 7.33(7.14, 7.52) | | | 3.73(3.58, 3.87) | 2.70(2.63, 2.77) | | | 13.19(13.09, 13.29) | | | 281.90(276.99, 286.81) |
| 12-14 y | | | 499 | | | 7.07(6.89, 7.26) | | | 3.83(3.68, 3.98) | 2.46(2.38, 2.53) | | | 13.64(13.49, 13.79) | | | 264.17(257.97, 270.37) |
| 15-17 y | | | 485 | | | 7.22(6.99, 7.46) | | | 4.15(3.95, 4.34) | 2.27(2.18, 2.35) | | | 14.09(13.91, 14.28) | | | 252.43(247.24, 257.63) |
| 18-24 y | | | 781 | | | 7.58(7.34, 7.81) | | | 4.46(4.29, 4.63) | 2.29(2.22, 2.35) | | | 14.21(14.09, 14.34) | | | 253.09(247.70, 258.47) |
| 25-34 y | | | 1018 | | | 7.52(7.33, 7.70) | | | 4.43(4.29, 4.58) | 2.28(2.24, 2.33) | | | 14.02(13.94, 14.10) | | | 249.19(245.38, 252.99) |
| 35-44 y | | | 993 | | | 7.39(7.20, 7.58) | | | 4.33(4.19, 4.48) | 2.25(2.20, 2.31) | | | 13.94(13.82, 14.06) | | | 250.24(245.53, 254.95) |
| 45-54 y | | | 994 | | | 7.14(6.93, 7.34) | | | 4.17(4.01, 4.34) | 2.17(2.10, 2.23) | | | 14.10(13.99, 14.21) | | | 243.55(239.08, 248.03) |
| 55-64 y | | | 1036 | | | 6.96(6.75, 7.17) | | | 4.01(3.83, 4.18) | 2.16(2.12, 2.21) | | | 14.06(13.93, 14.20) | | | 233.63(228.96, 238.29) |
| 65-74 y | | | 712 | | | 6.97(6.77, 7.16) | | | 4.12(3.96, 4.27) | 2.03(1.97, 2.10) | | | 13.85(13.70, 14.00) | | | 224.46(218.48, 230.45) |
| ≥75 y | | | 298 | | | 7.36(7.12, 7.60) | | | 4.40(4.21, 4.60) | 2.08(1.99, 2.17) | | | 13.32(13.10, 13.53) | | | 221.75(212.88, 230.63) |

Note: data was presented as means (95% credibility intervals).
