# Supplementary material for: Prevalence of neutropenia in US residents: a population based analysis of NHANES 2011–2018
Source: BMC Public Health. 2023 Jun 28;23:1254. doi: 10.1186/s12889-023-16141-5 (PMC10308693; doi:10.1186/s12889-023-16141-5)
Supplement: Supplementary file 3 — Supplementary Material 3 [file 12889_2023_16141_MOESM3_ESM.docx]

**Table S4. Number and Percentage of Males and Females with Neutropenia**

| **Age and sex group** | **Participants** | | | |
| --- | --- | --- | --- | --- |
|  | **Black** | **White** | **Mexican** | **Other** |
| **Males** |  |  |  |  |
| 1-2 y | 32(19.8)[13.8-25.8] | 25(12.1)[7.3-16.9] | 10(7.0)[3.0-11.1] | 8(4.3)[1.2-7.5] |
| 3-5 y | 24(9.8)[6.5-13.1] | 3(1.3)[0.0-2.8] | 3(1.5)[0.0-3.6] | 11(4.6)[1.9-7.3] |
| 6-8 y | 26(10.1)[5.9-14.2] | 4(1.2)[0.0-2.4] | 1(0.5)[0.0-1.4] | 5(1.8)[0.2-3.5] |
| 9-11 y | 36(14.2)[9.0-19.3] | 6(3.0)[0.5-5.6] | 4(2.4)[0.0-5.0] | 4(1.2)[0.0-2.5] |
| 12-14 y | 40(17.6)[11.3-24.0] | 5(1.9)[0.0-4.0] | 5(2.7)[0.3-5.0] | 5(1.8)[0.1-3.5] |
| 15-17 y | 28(12.1)[7.3-17.0] | 1(0.2)[0.0-0.5] | 3(2.5)[0.0-5.5] | 6(2.1)[0.4-3.7] |
| 18-24 y | 27(7.7)[4.5-10.9] | 0(0.0)[0.0-0.0] | 0(0.0)[0.0-0.0] | 1(0.2)[0.0-0.5] |
| 25-34 y | 10(3.0)[1.0-5.0] | 1(0.4)[0.0-1.1] | 1(0.4)[0.0-1.1] | 1(0.3)[0.0-0.7] |
| 35-44 y | 12(4.2)[1.8-6.7] | 3(0.6)[0.0-1.6] | 0(0.0)[0.0-0.0] | 1(0.2)[0.0-0.5] |
| 45-54 y | 11(2.9)[1.1-4.6] | 1(0.1)[0.0-0.2] | 2(0.6)[0.0-1.5] | 1(0.2)[0.0-0.5] |
| 55-64 y | 11(2.0)[0.7-3.3] | 3(0.6)[0.0-1.3] | 0(0.0)[0.0-0.0] | 6(1.2)[0.1-2.3] |
| 65-74 y | 10(3.0)[1.1-5.0] | 0(0.0)[0.0-0.0] | 0(0.0)[0.0-0.0] | 0(0.0)[0.0-0.0] |
| ≥75 y | 5(2.7)[0.3-5.2] | 2(0.2)[0.0-0.5] | 0(0.0)[0.0-0.0] | 1(1.0)[0.0-2.8] |
| **Females** |  |  |  |  |
| 1-2 y | 28(17.3)[11.1-23.6] | 9(5.1)[1.2-9.0] | 7(4.6)[1.5-7.6] | 13(8.8)[3.4-14.2] |
| 3-5 y | 20(10.9)[6.8-15.0] | 5(2.2)[0.2-4.3] | 6(3.7)[0.5-6.9] | 3(1.2)[0.0-2.6] |
| 6-8 y | 19(8.8)[3.2-14.3] | 2(0.7)[0.0-1.8] | 0(0.0)[0.0-0.0] | 3(2.9)[0.0-7.9] |
| 9-11 y | 31(10.8)[6.9-14.7] | 6(2.4)[0.3-4.4] | 1(0.4)[0.0-1.3] | 2(0.8)[0.0-2.0] |
| 12-14 y | 21(9.7)[5.4-13.9] | 2(0.8)[0.0-2.0] | 2(0.8)[0.0-2.0] | 2(0.7)[0.0-1.6] |
| 15-17 y | 7(3.7)[1.1-6.3] | 3(1.2)[0.0-2.8] | 0(0.0)[0.0-0.0] | 1(0.2)[0.0-0.5] |
| 18-24 y | 10(3.2)[0.6-5.8] | 0(0.0)[0.0-0.0] | 0(0.0)[0.0-0.0] | 2(0.7)[0.0-1.9] |
| 25-34 y | 13(3.5)[1.6-5.3] | 2(0.3)[0.0-0.7] | 1(0.4)[0.0-1.2] | 2(0.3)[0.0-0.7] |
| 35-44 y | 15(4.3)[2.1-6.6] | 2(0.3)[0.0-0.8] | 2(0.5)[0.0-1.1] | 0(0.0)[0.0-0.0] |
| 45-54 y | 12(3.1)[1.3-4.8] | 1(0.6)[0.0-1.7] | 0(0.0)[0.0-0.0] | 5(0.9)[0.1-1.6] |
| 55-64 | 15(3.0)[1.4-4.7] | 4(0.7)[0.0-1.4] | 1(0.6)[0.0-1.7] | 5(1.2)[0.3-2.1] |
| 65-74 | 6(1.9)[0.3-3.5] | 2(0.5)[0.0-1.2] | 1(0.5)[0.0-1.6] | 3(0.7)[0.0-1.6] |
| ≥75 | 3(1.5)[0.0-3.1] | 5(0.6)[0.1-1.0] | 0(0.0)[0.0-0.0] | 1(0.4)[0.0-1.1] |

Note: data was presented as number (percentage) (95% credibility intervals).
