# Supplementary material for: Prevalence of neutropenia in US residents: a population based analysis of NHANES 2011–2018
Source: BMC Public Health. 2023 Jun 28;23:1254. doi: 10.1186/s12889-023-16141-5 (PMC10308693; doi:10.1186/s12889-023-16141-5)
Supplement: Supplementary file 4 — Supplementary Material 4 [file 12889_2023_16141_MOESM4_ESM.docx]

**Table S3.** **Distribution of Population Grouped by Leukocyte Count and Neutrophil Count**

|  | **Leukocyte count, 10^9^ cells/L** | | | | | | | | | | |
| --- | --- | --- | --- | --- | --- | --- | --- | --- | --- | --- | --- |
| **Ethnic groups** | **<3** | **3-4** | **4-5** | **5-6** | **6-7** | **7-8** | **8-9** | **9-10** | **10-11** | **11-12** | **≥12** |
| **Black, n (%)** | 12(0.14) | 120(1.77) | 606(8.53) | 1292(17.39) | 1642(21.25) | 1437(17.24) | 1131(13.77) | 722(8.54) | 432(5.13) | 252(2.78) | 321(3.46) |
| **White, n (%)** | 28(0.68) | 259(5.20) | 785(15.94) | 1037(21.43) | 944(19.04) | 701(14.62) | 458(9.81) | 261(5.64) | 150(3.31) | 99(2.14) | 103(2.19) |
| **Mexican American, n (%)** | 2(0.06) | 33(1.01) | 183(6.29) | 423(13.25) | 667(21.73) | 603(19.46) | 478(15.74) | 303(10.34) | 153(5.02) | 89(3.21) | 120(3.88) |
| **Other races, n (%)** | 12(0.15) | 124(1.85) | 512(8.07) | 1078(18.33) | 1252(20.71) | 1076(18.83) | 786(13.23) | 442(8.11) | 260(4.82) | 130(2.80) | 160(3.10) |
|  | **Neutrophil count, 10^9^ cells/L** | | | | | | | | | | |
| **Ethnic groups** | **<1** | **1-2** | **2-3** | **3-4** | **4-5** | **5-6** | **6-7** | **7-8** | **8-9** | **9-10** | **≥10** |
| **Black, n (%)** | 8(0.07) | 91(1.02) | 148(2.39) | 1093(15.11) | 2080(27.63) | 1948(23.51) | 1306(15.46) | 700(8.16) | 339(3.79) | 173(1.92) | 81(0.93) |
| **White, n (%)** | 15(0.37) | 22(0.48) | 559(11.42) | 1323(27.19) | 1248(25.86) | 819(16.76) | 398(8.39) | 234(5.14) | 124(2.69) | 50(0.97) | 33(0.72) |
| **Mexican American, n (%)** | 1(0.03) | 25(0.80) | 53(1.58) | 376(12.80) | 824(26.76) | 828(26.31) | 483(15.52) | 243(8.70) | 127(4.39) | 64(2.14) | 30(0.98) |
| **Other races, n (%)** | 4(0.05) | 37(0.58) | 187(2.76) | 1021(16.99) | 1737(28.96) | 1390(23.95) | 770(13.30) | 369(6.67) | 175(3.94) | 102(2.02) | 40(0.77) |

Note: data was presented as number (percentage).
